# Quantitative Characterization of Olaparib in Nanodelivery System and Target Cell Compartments by LC-MS/MS

**DOI:** 10.3390/molecules24050989

**Published:** 2019-03-11

**Authors:** Roberta Ottria, Alessandro Ravelli, Matteo Miceli, Sara Casati, Marica Orioli, Pierangela Ciuffreda

**Affiliations:** 1 Dipartimento di Scienze Biomediche e Cliniche “Luigi Sacco”, Università degli Studi di Milano, Via G.B. Grassi 74, 20157 Milano, Italy; roberta.ottria@unimi.it (R.O.); matteo.miceli@unimi.it (M.M.); 2 Dipartimento di Scienze Biomediche, Chirurgiche ed Odontoiatriche, Sezione di Tossicologia Forense, Università degli Studi di Milano, Via Mangiagalli 37, 20133 Milano, Italy; alessandro.ravelli@unimi.it (A.R.); sara.casati@unimi.it (S.C.); marica.orioli@unimi.it (M.O.)

**Keywords:** Olaparib, Olaparib nano-formulation, HPLC, bioanalysis, mass spectrometry, sample preparation

## Abstract

Olaparib, an orally active inhibitor of poly(ADP-ribose)polymerase(PARP), is the drug of choice in the treatment of gBRCA1/2+ metastatic breast cancers. Unfortunately, Olaparib is poorly soluble with low bioavailability and tumor accumulation; nano-delivery could be a good choice to overcome these disadvantages. Here, a rapid and robust HPLC-ESI–MS/MS method for the quantification of Olaparib in ferritin nano-carriers led to the development of cells compartments, different tissues, plasma and urines and were validated to assess the effects of nano-delivery on cell compartment distribution of the drug. This method allows the quantification of Olaparib within the linear range of 0.1–10ng/mL in cells culture medium and cell cytoplasm, of 0.5–10ng/mL in nuclei, of 0.5–100ng/mL in plasma and urine and of 10–500ng/mL in tissue samples (kidney and liver). The limit of quantification was found to be 1.54 ng/mL for liver, 2.87 ng/mL for kidney, and lower than 0.48 ng/mL for all matrices. The method has been applied to quantify Ola encapsulated in ferritin-nano-carriers during the nano-drug development. The application of the method to human BRCA-mutated cell model to quantify the Olaparib distribution after incubation of free or ferritin-encapsulated Olaparib is also reported. This sensitive method allows the quantification of low concentrations of Olaparib released from nano-carriers in different cell compartments, leading to the determination of the drug release and kinetic profile of an essential parameter to validate nano-carriers.

## 1. Introduction

Poly(ADP-ribose) Polimerase (PARP) plays an important role in a number of DNA repair pathways. PARP inhibitors (PARPi) such as Olaparib (Ola) exploit the concept of synthetic lethality by selectively targeting cancer cells with defective DNA repair pathways. Ola is a poorly soluble drug and is currently administered orally, which results in a limited bioavailability, poor accumulation in tumors, and systemic toxicity. In order to overcome Ola’s disadvantages, we collaborated to develop a novel “Nano-Olaparib,” which specifically targets the tumor, providing a safe vehicle for intravenous delivery and increasing the drug bioavailability. This nano-cage is also able to mediate the direct delivery of drugs into the nuclear compartment through a self-triggered mechanism, as reported for H-Ferritin loaded with Doxorubicin [1].

Having a good assay method is important to optimize the formulation of Ola-loaded nanoparticles and to study in vitro release profiles during the development of drug delivery systems, and to determine drug distribution in target and non-target organs or cells compartments. Several clinical trials, containing HPLC/MS-MS Ola quantification for pharmacological studies, have been published, but no technical details on analytical method employed were given [2,3,4]. In the last decade, a few methods for Ola quantification in human plasma (100 uL), characterized by a calibration range of 10–5000 ng/mL [5,6] and high limit of quantification (10 ng/mL) or expensive and time consuming solid phase extraction procedure [7] were published. Interestingly, Roth and coworkers developed an HPLC/MS-MS method for Ola quantification in human plasma with large applicability to clinical trials due to its high sensitivity (0.5 ng/mL) and a large range of calibration (0.5–50000 ng/mL) [8]. Only a very recent article presents an HPLC coupled with Ultraviolet Diode Array Detector (HPLC-UV-DAD) method for Ola quantification inside cancer cells [9].

The aim of this study is to develop and validate a liquid chromatography–tandem mass spectrometry (LC-MS/MS) method to quantify Ola in different bio-matrices as a powerful tool for Ola nano-formulation optimization and drug distribution evaluation for pharmaceutical research. The quantification of the drug inside the nano-delivery system is fundamental to not only the loading process optimization, but also to characterize nano-formulation and set doses for pharmacological treatment. Of particular novelty and importance is also the analysis of cytoplasmic and nuclear fractions content of the drug. The quantification of Ola in these cell fractions is crucial for drug delivery studies to demonstrate the release of the drug in the target cellular space. Ola’s target is indeed the nuclear PARP and the availability of an analytical method able to assess and quantify nuclear accumulation of the drug becomes fundamental for delivery systems set up, as well as to verify the drug mechanism of action. For this reason, a simple and selective method for Ola quantification in Ola-loaded HFn nanoparticles and different cell compartments was proposed. This method allows, for the first time, the quantification of Ola encapsulated in nanoparticles for delivery, an essential information for the set-up of a drug delivery system and for the planning of pharmacological experiments. Moreover, the quantification of the drug in different cell compartments is a critical challenge to study drug accumulation in target compartments and drug mechanism of action. Therefore, this method has been applied on BRCA-mutated and sporadic triple negative breast cancer to assess how encapsulation modifies drug intracellular distribution [10].

## 2. Results

### 2.1. Instrument Parameters 

To improve the responses of the target compounds and to reduce the analysis time, different reverse phase columns and isocratic or gradient methods for products elution have been assessed. Phenomenex Gemini C18 column, coupled with a gradient of acetonitrile and ammonium formate 10 mM (0,1% formic acid) as mobile phases, exhibited the best sensitivity and peak shape. The retention times of Ola and Ola-d8, 4.53 min and 4.54 min respectively, were low enough to allow a short total runtime of 8.0 min comprising cleaning and reconditioning of the column. Mass parameters were optimized by infusing an Ola solution at the concentration of 100 ng/mL in methanol, acquiring both in the positive and negative ionization mode: Positive ionization mode gave the better signal response. The source/gas and compound parameters were optimized to obtain the highest ion abundance of the peak relative to [Ola-H]+ (*m*/*z* 435.4) and [Ola-d8 -H]+ (*m*/*z* 443.4). Collision energy was varied from 0 to 60 V to obtain the best response for the following product ions: *m*/*z* 367.7 and *m*/*z* 281.3 (28 V) for Ola and *m*/*z* 375.7 and *m*/*z* 281.3 (45 V) for Ola-d8, used for quantitative MRM analysis. Transitions *m*/*z*: 435.4 ≥ 367.7 and *m*/*z*: 443.4 ≥ 375.7 were selected for the quantification of Ola and Ola-d8, respectively.

### 2.2. Sample Preparation

**Figure 1:**
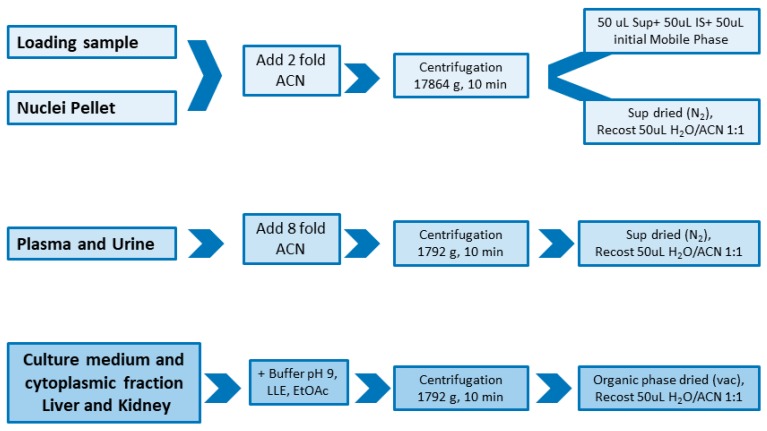
Graphical overview of sample purification and extraction methods applied to all bio-matrices.

Ola-loading samples and cell nuclei samples were purified by a simple protein precipitation step with acetonitrile using 200% *v*/*v* in respect to sample volume. Additionally, urine and plasma samples gave clean chromatograms, applying only protein precipitation step using 800% *v*/*v* cooled acetonitrile in respect to sample volume. Instead, cell culture medium, cell cytoplasmic compartments and tissues media require liquid–liquid extraction with ethyl acetate after dilution of the sample with the pH9 buffer solution. Figure 1 reports a graphical overview of sample purification and extraction methods applied to all bio-matrices.

### 2.3. Method Validation

Specificity of the method was achieved by the selection of a precursor ion followed by detection and quantification of product ions. All reagents and disposables used for the set-up of the method did not interfere with the detection or quantification of Ola. False positive response or co-eluting components were not detected in analyzed bio-matrices and no carryover was observed.

#### 2.3.1. Linearity, LOD and LOQ

The calibration curves showed excellent linearity (r > 0.996) over the following concentration ranges: 25–750 ng/mL for phosphate buffer (matrix for loading samples), 10–500 ng/mL for tissue matrices, 0.5–10 ng/mL nuclei pellet, 0.1–10 ng/mL for cytoplasmic fraction and 0.5–100 ng/mL for plasma and urine. LOD and LOQ levels have been calculated for all bio-matrices and are listed in Table 1 (LOD 0.02–0.86 ng/mL and LOQ 0.09–2.87 ng/mL). All the assay values were found to be within the accepted variable limits (±15% RSE, ≤15% CV).

#### 2.3.2. Precision and Accuracy

The intra, inter-day precision, and accuracy were operated at three concentration levels. Intra-day precision ranges from 2.7% to 8.7%, inter-day from 4.7% to 9.8%, intra-day and inter-day accuracy range from 4.8% to 11.1% and from 6.9% to 13.4%, respectively. The absolute values were within 15%, therefore precision and accuracy were acceptable (Table 2). Additionally, the intra, inter-day precision, and accuracy for diluted samples were determined for plasma and urine matrices and loading samples analyzing QC samples after a dilution step. LQC2, MQC2, and HQC2 represent plasma, urine, or loading samples diluted 50 fold, 100 fold, and 200 fold, respectively, and analyzed over three batch runs. Additionally, for diluted samples, absolute values were acceptable (%CV ≤ 15%, %SE ≤ 15%, Table 2).

#### 2.3.3. Recovery and Matrix Effect

The mean extraction recoveries determined using three replicates of QC at three concentration levels (LQC, MQC, and HQC) in all bio-matrices were shown in Table 1. The recovery ranges from 75% to 81% in plasma and from 58% to 85% in tissues matrices, while for all the other matrices, it is higher than 85%. To determine the matrix effect, that may impact on HPLC-MS analysis, the relative peak areas obtained after spiking the extracted samples at the three concentration levels were compared to similarly prepared aqueous standard solutions. The results showed that ion suppression or enhancement due to different matrix ranges from −21% to +36% (Table 1). 

#### 2.3.4. Stability

The Stability of Ola in Bio-Matrices Was Fully Evaluated under Various Conditions, by Analyzing Six Replicates of QC Samples at LQC and HQC Concentration Levels. The Results were Within ±15%. 

### 2.4. Application of the Method

**Figure 2:**
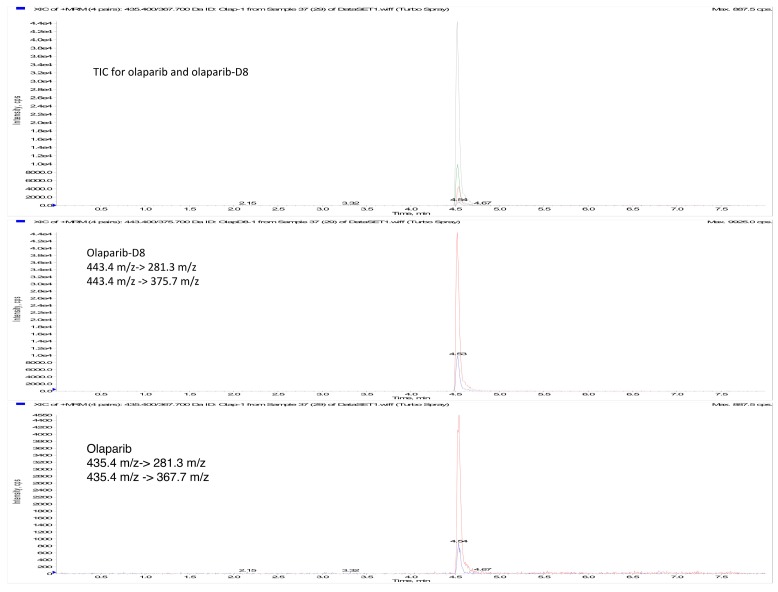
Representative chromatogram of cytoplasmic extract (0.2 ng/mL Ola). From the top: Total ion Chromatogram (MRM signals are overlayed), Ola-d8 extraction, and Ola extraction.

**Figure 3:**
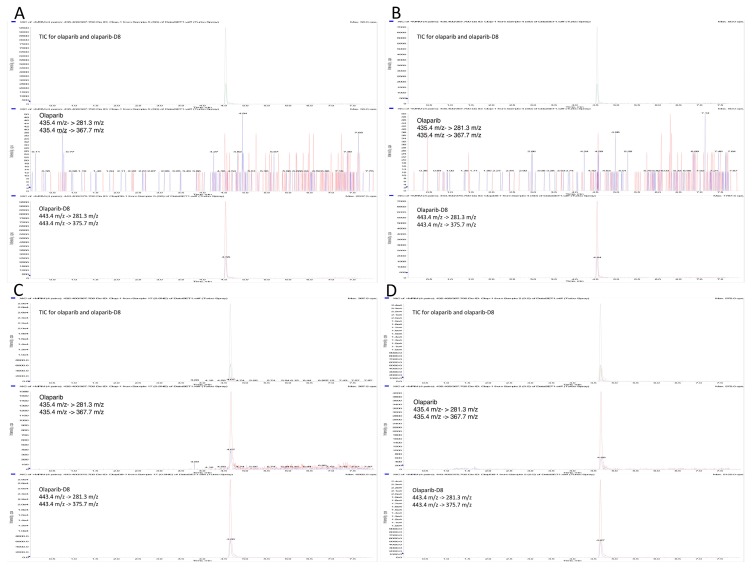
Representative chromatograms of validation samples: (**A**) Blank nuclei, (**B**) blank Cytoplasm, (**C**) nuclei extract at LOQ (0.42 ng/mL), and (**D**) cytoplasmic extract at LOQ (0.10 ng/mL). In all figures from the top: Total ion Chromatogram (MRM signals are overlayed), Ola extraction, and Ola-d8 extraction.

The present analytical method was applied to develop a new delivery system for Ola, two different loading method were assessed to introduce the drug in ferritin nano-carriers, as described elsewhere [10]. The first method, that plan the opening of the nano-carrier and its re-assembly at different pH values gave Ola concentrations of 240 ± 40 ng/mL (mean ± sd, 15 loading assay), while the second method, that plan the Ola complexation with copper before loading, gave Ola concentrations of 1117 ± 816 ng/mL (mean ± sd, 10 loading assay). Since the second loading method allows to encapsulate high quantities of the drug in the nano-carrier leading to Ola concentrations higher than the calibration range of the method (25–750 ng/mL), accuracy and precision were assessed also on LQC2, MQC2, and HQC2, loading samples diluted 50 fold, 100 fold, and 200 fold (Table 2). The method was also successfully used to determine the amount of Ola in both cell culture media and lysates after incubation of free or H-Fn encapsulate Ola [10]. Figure 2 and Figure 3 report chromatograms relative to a real sample of cytoplasmic extract and two matrices, blank, and spiked at the LOQ (cell nuclei and cytoplasmic fraction), respectively. 

**Figure 4:**
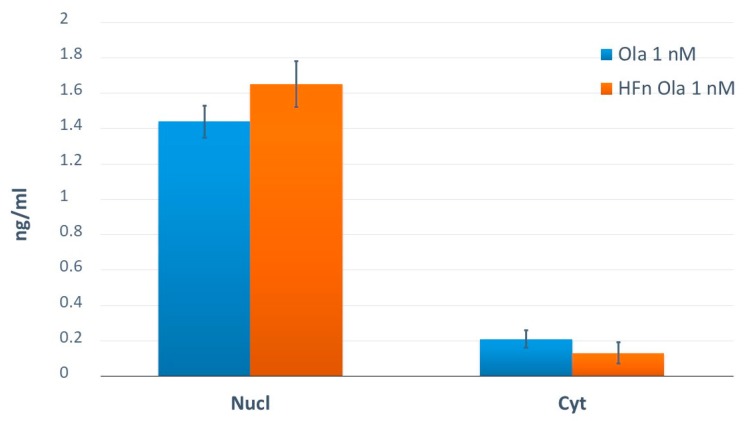
Application of the method. Concentrations expressed in ng/mL of nuclei and cytoplasmic fraction of HCC1937 cells treated with free or encapsulated Ola.

Figure 4 reports measured concentrations in nuclei and cytoplasmic fraction of HCC1937 cells treated with free or encapsulated Ola.

## 3. Discussion

The development and validation of a new HPLC/MS-MS analytical method for the quantification of Ola in different bio-matrices is here reported. As described previously, to date, a few methods for plasma quantification of Ola for clinical application have been reported in literature. The present method is comparable with that published by Roth and coworkers [8], the highest sensitive and robust method to date developed, in terms of sensitivity, calibration range, and recovery of the analyte. Interestingly, the present method allows to reach very good performance in sensitivity, accuracy, and reproducibility starting from 25 μL of plasma matrix, instead of 100 μL, leading to a method suitable not only for clinical studies, but also for pharmacological applications on animal models. Only recently, Daumar and coworkers present, for the first time, the Ola quantification inside the cancer cells developing an HPLC-UV-DAD method able to quantify Ola in the entire cell in the calibration range of 200–2000 ng/mL [9]. The present method, based on the liquid chromatography tandem mass technique, the method of choice for the detection and quantification of low drug levels, allows to discriminate between the cytoplasmic and the nuclear contents of the drug and to quantify very low amounts of OLA, until 0.1 ng/mL. Moreover, the addition of urine and liver and kidney tissues renders this analytical method versatile and of large applicability to clinical and pharmacological research to investigate the Ola washout. Finally, a method for the quantification of the Ola in nano-delivery systems is here reported for the first time.

### 3.1. Sample Preparation

A simple method of protein precipitation with acetonitrile, followed by centrifugation, has been set-up for sample purification. Indeed, culture medium and cytoplasmic extract and tissue samples required a liquid-liquid extraction, which allows extracts concentration, in order to obtain good recovery and purification, suitable for quantification of a very low amount of Ola.

### 3.2. Method Validation

The assay passed the validation following the acceptance criteria of FDA guidelines [11] in all the different bio-matrices. For the method, good selectivity was achieved by the selection of a precursor ion for Ola and Ola-d8 followed by the quantification of product ions in MRM mode. All reagents and disposables used for the sample treatment and analyses did not interfere with the detection or quantification of Ola. Moreover, false positive response or co-eluting components were not detected and no carryover was observed.

In order to have a versatile method appropriate also for clinical application accuracy and precision were determined also for diluted samples for plasma and urine matrices analyzing QC samples after a dilution step. The sample treatment and extraction procedures applied allow to obtain good recoveries from all bio-matrices assessed giving, together with the LOQ values, a method suitable for the analyses of small amounts of samples containing low quantities of the drug. Relevant ion suppression, displayed by the analyses performed in nuclei pellet and plasma matrices, underline the major complexity of these matrices that lead to less clean extracts. Despite the significant values of matrix effects obtained, the use of pooled matrices, that allows to estimate a mean matrix effect characteristic of the matrix (from 10 different batches), of Ola-d8 as internal standard and the low values of LOQ obtained allow to overcome possible effects due to a single sample on quantification leading to a fit-for-purpose method. 

Moreover, stability experiments indicate that Ola is stable at room temperature for 15 h, at −80 °C for 30 days, and after three freeze-thaw cycles.

## 4. Materials and Methods 

### 4.1. Chemicals

Ola and Ola-d8 were purchased from AstraZeneca (London, UK). HPLC-MS grade solvents and ethyl acetate were purchased from Sigma Aldrich (St. Louis, MO, USA), and Buffer pH 9 from Panreac Química SLU (Barcellona, Spain).

### 4.2. Instruments

The LC/MS-MS analysis was performed with a Dionex Ultimate 3000 HPLC system (ThermoFischer, Waltham, MA, USA), interfaced to a 4000 QTRAP (ABSciex S.r.l., Milano, Italy). The mass spectrometer operated in positive electrospray ionization (ESI) mode with capillary voltage at 5.5 kV. Analyst and Multiquant software (ABSciex S.r.l., Milano, Italy) were used for instrument control and data analysis. The separation of Ola and Ola-d8 was performed on a Phenomenex Gemini C18 column (2.1 × 150 mm, 3 um) protected by a Phenomenex precolumn C18 cartridge (4 × 3.0 mm). Mobile phase was 10 mM ammonium formiate and 0.1% formic acid in water (A) and acetonitrile (B). The separation was optimized using the following 8 min gradient: 0–0.5 min A 95%, B reached 100% in 4 min and was maintained for 1.5 min. The column was reconditioned with A 95% for 3.5 min. The flow rate was 0.4 mL/min and the injection volume was set to 20 μL. Nitrogen was used as the de-solvation gas at a pressure of 40 psi and the capillary temperature was 550 °C.

### 4.3. Standard Solutions

Working solutions for Ola (10 μg/mL, 1 μg/mL and 100 ng/mL) and Ola-d8 (100 ng/mL) were prepared by dilution of the standard reference solutions prepared dissolving 1mg of Ola or Ola-d8 in 1 mL of methanol and stored at −20 °C until use.

### 4.4. Control Plasma, Urine, and Mouse Tissue, Cell Extracts and Ola Loading Samples Collection

#### 4.4.1. Ola Loading Samples

A solution of Ola (1 mg/mL in NaOH 1M) was added to a solution of H-Ferritin (0.15 mM NaCl) and different loading procedures have been assessed, as described elsewhere [10]. Encapsulated-Ola was separated from free Ola by a gel filtration to Sephadex G-25 column and the resultant solution used for Ola quantification.

#### 4.4.2. Cells Samples

Cell fractions of untreated HCC1937 cells, cultured as for H-Fn Ola or free Ola treatment experiments, were used as standard matrices for method setup. Cell nuclei samples and cytoplasmic extracts were obtained as described before [8]. Briefly, HCC1937 cells were seeded in a 6-multiwell plate at 1 × 10^6^ cells/well and incubated at 37 °C for 24 h then cells were harvested with Trypsin/EDTA and centrifuged 5 min at 896 g. Pellets were washed twice with phosphate buffer, suspended in 1 mL of Nuclei Extraction Buffer (10 mM Hepes, pH 7.4, 320 mM Sucrose, 5 mM MgCl, 1%Triton X-100) and incubated on ice for 10 min. 5 min centrifugation at 896 g was applied and two washing cycles with 1 mL of Nuclei Wash Buffer (10 mM Hepes, pH 7.4, 320 mM Sucrose, 5 mM MgCl) were performed. Nuclei pellets were isolated and the supernatant was used as a matrix for cytosolic extract experiments. The purity of nuclei and cytoplasmic fractions was checked by Western blot analysis, as described before [10].

#### 4.4.3. Plasma and Urines

Control human plasma and urine samples were obtained from healthy volunteer colleagues, and all volunteers gave informed consent to offer their biological samples for research intent. Blood was collected into a vial containing K^+^-EDTA and immediately centrifuged. Aliquots of 10 mL of pooled plasma were stored at −80 °C. Human urines were collected after a circadian cycle and 10 mL aliquots of pooled urines were stored at −80 °C. 

#### 4.4.4. Tissue Samples

For the set up and validation studies, aliquots of tissue (kidney and liver) obtained from healthy BALB/c mice from previous studies [12] were used. Mice were sacrificed and organs were explanted, weighted, transferred in a polypropylene plastic tube. Organs immediately frozen by liquid nitrogen immersion and stored at −80 °C. Whole organs were then homogenized in water (10% *w*/*v*) with potter (Glas-Col homogenizer) and divided in aliquots of 200 μL and stored at −80 °C.

### 4.5. Calibration Curves

#### 4.5.1. Ola Loading Samples

For each calibration point, the proper volume of Ola working solution (1 μg/mL or 10 μg/mL) was selected to reach the following final concentrations in phosphate buffer: 0, 25, 50, 100, 250, 500, and 750 ng/mL. 10 μL of calibration solutions spiked with Ola-d8 (100 ng/mL working solution) were further diluted in 0.98 mL of mobile phase (10 mM ammonium formiate and 0.1% formic acid/acetonitrile 95/5 *v/v*) and directly injected for analysis.

#### 4.5.2. Cell Nuclei Samples

An aliquot of Ola-untreated pooled pellet (about 5 × 10^5^ cell nuclei) was reconstituted in 2 mL of LC-MS water and 100 μL of this mixture were used as blank matrix for the calibration set-up. Calibration points (0-0.5-1-2.5-5-10 ng/mL) were spiked with Ola-d8 (100 ng/mL working solution), purified as described in paragraph 4.6.2, dried, and after reconstitution in 50 μL water/acetonitrile 1/1 *v*/*v* was analyzed.

#### 4.5.3. Culture Medium and Cytoplasmic Fraction

Supernatant obtained from nuclei extraction of Ola-untreated cells, containing cell cytosol, was used as a matrix for calibration curves relative to cytoplasmic extract. Calibration points (0, 0.1, 0.5, 1, 2.5, 5, and 10 ng/mL) were spiked with Ola-d8 (100 ng/mL working solution), extracted, and reconstituted, as described in sample preparation section and analyzed.

#### 4.5.4. Plasma and Urine Samples

Calibration Standard (CS) samples were prepared in plasma and urines (25 μL) by adding different volumes of Ola working solution to reach final concentrations of 0, 0.5, 1, 2.5, 5, 10, 25, 50, and 100 ng/mL. CS were spiked with Ola-d8 (100 ng/mL working solution), extracted, and reconstituted, as described in the sample preparation section and analyzed. 

#### 4.5.5. Tissue Samples

Calibration points (10, 25, 50, 100, 250, and 500 ng/mL) were prepared in tissue homogenizate (200 μL 10% *w*/*v*), samples were spiked with Ola-d8 (100 ng/mL working solution), extracted, and reconstituted, as described in sample preparation section and analyzed.

#### 4.5.6. Quality Control (QC) Samples

QC samples at low (LQC), medium (MQC) and high (HQC) concentration levels were prepared in all matrices. Each sample was spiked with different volumes of Ola and Ola-d8 working solution, and extracted as described in sample preparation section. LQC, MQC and HQC levels were set at 25, 100, and 500 ng/mL for loading samples, at 10, 100, and 500 ng/mL for tissue, at 0.5, 2.5, and 10 ng/mL for nuclei, culture medium, and cytoplasmic extract samples, and at 0.5, 5, and 50 ng/mL for plasma and urines.

### 4.6. Sample Preparation

#### 4.6.1. Ola Loading Samples

100 μL of cold acetonitrile were added to 50 μL of HFn-Ola solution obtained from Ola loading procedure for H-Ferritin precipitation and Ola release. Samples were centrifuged at 17,864 g for 10 min, 50 μL of the supernatant and 5 μL of Ola-d8 (100 ng/mL working solution) were diluted in 0.445 mL of mobile phase and 20 μL were injected for analysis.

#### 4.6.2. Cell Nuclei Samples

Nuclei pellets were suspended in 100 uL of water (obtained from 5 × 10^5^ cells) and 200 μL of cold acetonitrile were added, solution was spiked with Ola-d8 (100 ng/mL working solution) and centrifuged at 17,864 g for 10 min. The supernatant was dried under nitrogen, reconstituted with 50 μL of water/acetonitrile 1/1-*v*/*v* and analyzed.

#### 4.6.3. Culture Medium, Cytoplasmic Fraction and Tissue Samples

The samples (100 μL of cell culture medium or cytoplasmic fraction or 200 μL of tissue (liver or kidney) homogenized in water (10% *w*/*v*)) were purified by a liquid-liquid extraction with 2 mL of ethyl acetate, after the addition of pH 9 buffer and Ola-d8 (100 ng/mL working solution). Samples were centrifuged at 1792 g for 10 min, supernatant was dried and reconstituted with 50 μL of water/acetonitrile 1/1-*v*/*v* and analyzed.

#### 4.6.4. Plasma and Urine Samples

25 μL of plasma or urine were spiked with Ola-d8 (100 ng/mL working solution), 200 μL of cold acetonitrile were added and the obtained solution was centrifuged at 1792 g for 10 min. The supernatant was dried under nitrogen, reconstituted in 50 μL of water/acetonitrile 1/1-*v*/*v* and analyzed.

### 4.7. Sample Preparation

The method was validated according to the US Food and Drug Administration (FDA) guidelines [11].

#### 4.7.1. Sensitivity and Carry-Over

Reagents and/or disposable blank water were extracted, following the procedures described before and the extracts analyzed in triplicate to evaluate and exclude interferences and false positive responses derived from sample preparation. The carry-over was evaluated by analyzing a blank solution (water/acetonitrile 50/50-*v*/*v*) immediately after the injection of the highest CS. The Signal-Noise ratio relative to Ola peak was <3.0. Sensitivity was expressed by LOD and LOQ calculated on calibration curves prepared in the different bio-matrices. LOD and LOQ are expressed, respectively, as 3.3 and 10 times the ratio between the standard deviation of the response and the slop of the calibration curve. LOD and LOQ values calculated for all bio-matrices are reported in Table 1. 

#### 4.7.2. Linearity, Precision, and Accuracy

The linearity was assessed on the different bio-matrices over their respective calibration range from three batches of analytical runs. Different calibration ranges for the bio-matrices have been chosen in relation to concentrations expected in real samples. Linear calibration curves were built by the ratio of analyte peak area/internal standard peak area and determination and variation coefficients (r2 and CV) were calculated. Precision and accuracy were determined by the analysis of QC samples at three concentration levels LQC, MQC and HQC over three batch runs. For each QC, analysis was performed in six replicates on each day. Precision is denoted by percent coefficient of variance (% CV) while accuracy is denoted by a percent relative standard error (% RSE). The accuracy and precision were required to be within ±15% RSE of the nominal concentration and ≤15% CV (Table 2).

#### 4.7.3. Recovery and Matrix Effect

Absolute recovery was evaluated in all the different bio-matrices by comparing the mass spectrometric response of the LQC, MQC, and HQC to that of extracted blank matrices. These samples were spiked at LQC, MQC and HQC of Ola after the extraction procedure. All samples were analyzed in six replicates and the overall absolute recovery was expressed as the ratio of Ola peak area of the two sets of samples. The average % RE was determined and the calculated precision (CV) did not exceed 15%. Matrix effects were studied by preparing LQC and HQC for the three matrices (pooled matrices from ten different wells) and for blank solution. In particular, 50 uL aliquot of matrices and blank water were extracted and spiked with a corresponding LQC and HQC of Ola after reconstitution in acetonitrile/water 50/50-*v*/*v*. The mean peak area of Ola determined in the blank water (no matrix effect) was compared with the mean area obtained in the three matrices. The gain or the reduction in the mean area measured in the three matrix samples was reported as ion enhancement/suppression percentage (Table 1).

#### 4.7.4. Sample Stability

The analyte stability in working solutions was evaluated at −80 °C for 1 month, at room temperature for 15 h, and after three freeze/thaw cycles, in triplicate. The mean values of the triplicate samples were compared with those of freshly prepared QC samples.

#### 4.7.5. Statistics

If not otherwise specified, quantitative analyses were performed in triplicate.

## 5. Conclusions

A rapid and accurate quantification method for Ola by HPLC-MS/MS from different biological complex matrices has been developed and validated. This method allows, for the first time, the quantification of Ola loaded in nano-carriers and in cell fractions as cytoplasmic compartments and nuclei pellet. The quantification of the drug inside nano-delivery systems is fundamental not only for the nano-drug development and optimization but also to characterize nano-formulation and is essential to set doses for pharmacological treatment. Moreover, the quantification of drugs in bio-matrices as different cell fractions is central to study and verify the supposed accumulation of the drug in its target cell compartment and the drug mechanism of action. This selective and sensitive method displayed a good linearity (r2 > 0.996), precision and accuracy in all the assessed bio-matrices. The liquid-liquid extraction, the short chromatographic run time (8 min including reconditioning), the validation of the method in different bio-matrices as cell compartments, mouse tissues ad plasma and urines, and in particular, the requirement of a very low volume of plasma and urine, render this method attractive for pharmacological research, both on cell or animal models. Moreover, the method is suitable for the analysis a large number of samples and allows the quantification of very low amounts of Ola. Finally, the method has been applied to pharmacokinetic studies of a new Ola nano-formulation based on ferritin nano-carriers [10].

## Figures and Tables

**Table 1:** Limit of the assay: LOD (Limit of Detection expressed in ng/mL) and LOQ (Limit of Quantification expressed in ng/mL). Recovery expressed as percentage and matrix effect expressed as a percentage of ion suppression (-) or enhancement.

			Matrix Effect		Recovery		
Matrix	LOD	LOQ	LQC	HQC	LQC	MQC	HQC
Cytoplasmic Fraction	0.04	0.10	36	31	88	87	90
Nuclei Pellet	0.21	0.48	−9	−17	85	88	92
Phosphate Buffer	0.02	0.09	5	11	91	96	93
Plasma	0.19	0.48	−15	−21	75	81	80
Urine	0.02	0.12	7	3	89	92	97
Liver	0.47	1.54	26	31	58	75	80
Kidney	0.86	2.87	32	28	63	82	85

**Table 2:** Intra- and inter-day precision (%CV) and accuracy (%RSE) of Ola in different bio-matrices for normal (LQC, MQC, and HQC) and diluted samples (LQC2, MQC2, and HQC2).

	(%CV) Intra-Day			(%CV) Inter-Day			(%RSE) Intra-Day			(%RSE) Inter-Day		
MATRICES	LQC	MQC	HQC	LQC	MQC	HQC	LQC	MQC	HQC	LQC	MQC	HQC
Phosphate Buffer	5.2	4.7	6.8	7.6	9.8	4.7	4.8	5.4	6.9	9.1	12.2	9.9
Cytoplasmic Fraction	5.6	7.6	4.3	8.8	6.6	5.4	8.6	7.1	7.2	11.7	11.3	9.8
Nuclei Pellet	4.4	3.2	6.1	4.7	6.6	5.8	11.1	6.3	8.1	12.2	8.5	7.5
Plasma	7.7	8.2	5.4	9.5	7.4	8.6	10.4	8.9	8.8	13.4	9.7	10.2
Urine	3.7	2.7	5.2	4.7	7.5	7.3	6.9	4.8	5.5	9.8	8.1	6.9
Liver	5.4	7.6	3.7	7.3	6.7	5.9	9.4	7.5	6.6	9.6	8.5	7.8
Kidney	8.7	6.2	5.7	6.2	6.5	7.3	8.9	8.1	8.9	11.2	11.8	10.8
	**LQC2**	**MQC2**	**HQC2**	**LQC2**	**MQC2**	**HQC2**	**LQC2**	**MQC2**	**HQC2**	**LQC2**	**MQC2**	**HQC2**
Plasma	9.9	8.1	6.7	10.1	8.1	8.9	10.1	9.9	8.5	13.8	10.4	9.9
Urine	4.8	5.2	6.1	5.5	6.8	6.5	6.7	6.8	7.8	10.5	8.5	6.7
Loading	5.7	5.2	2.3	4.5	4.3	4.5	3.6	4.6	2.9	5.1	3.8	4.2
