# Correction: Ottria, R., et al. Quantitative Characterization of Olaparib in Nanodelivery System and Target Cell Compartments by LC-MS/MS. *Molecules* 2019, *24*, 989

**DOI:** 10.3390/molecules24091771

**Published:** 2019-05-07

**Authors:** Roberta Ottria, Alessandro Ravelli, Matteo Miceli, Sara Casati, Marica Orioli, Pierangela Ciuffreda

**Affiliations:** 1 Dipartimento di Scienze Biomediche e Cliniche “Luigi Sacco”, Università degli Studi di Milano, Via G.B. Grassi 74, 20157 Milano, Italy; roberta.ottria@unimi.it (R.O.); matteo.miceli@unimi.it (M.M.); 2 Dipartimento di Scienze Biomediche, Chirurgiche ed Odontoiatriche, Sezione di Tossicologia Forense, Università degli Studi di Milano, Via Mangiagalli 37, 20133 Milano, Italy; alessandro.ravelli@unimi.it (A.R.); sara.casati@unimi.it (S.C.); marica.orioli@unimi.it (M.O.)

The authors wish to make the following corrections to this paper [1]: the authors wish to insert additional sentences in the Funding and Acknowledgments section.

**Funding:** Grant Cariplo 2016-0919 (Serena Mazzucchelli).

**Acknowledgments:** The authors thank Fabio Corsi and Serena Mazzucchelli for biological material and the University of Milan for financial support.

In the original version of our article [1], insufficient acknowledgement was given for the source of some biological matrices, the acknowledgements have been altered to more appropriately recognize support and funding. The authors would like to apologize for any inconvenience caused to the readers by these changes.
